# Genetics and sex influence peripheral and central innate immune responses and blood-brain barrier integrity

**DOI:** 10.1371/journal.pone.0205769

**Published:** 2018-10-16

**Authors:** Michelle A. Erickson, W. Sandy Liang, Elizabeth G. Fernandez, Kristin M. Bullock, Jarl A. Thysell, William A. Banks

**Affiliations:** 1 Geriatric Research, Education and Clinical Center (GRECC), Veterans Administration Puget Sound Healthcare System, Seattle, Washington, United States of America; 2 Department of Medicine, Division of Gerontology and Geriatric Medicine, University of Washington, Seattle, Washington, United States of America; 3 Department of Molecular and Medical Pharmacology, University of California Los Angeles, Los Angeles, California, United States of America; Case Western Reserve University, UNITED STATES

## Abstract

Lipopolysaccharide (LPS) is a stimulator of the innate immune system and is routinely used in animal models to study blood-brain barrier (BBB) dysfunction under inflammatory conditions. It is appreciated that both humans and mice have sexually dimorphic immune responses, which could influence the brain’s response to a systemic inflammatory insult. Mouse strain is also an important factor that can contribute to pathophysiological responses to inflammatory stimuli. Therefore, we aimed to test whether BBB disruption and the associated cytokine profiles in response to LPS differed in male and female mice from two mouse strains most commonly used in blood-brain barrier studies: CD-1 and C57BL6/J (C57). Mice were treated with saline, a single injection of 0.3, or 3mg/kg LPS, or three injections of 3mg/kg LPS, and studied 28 hours after the first LPS injection. To assay BBB disruption, we utilized the tracer 99mTc-DTPA. A 23-plex panel of cytokines was assayed in brain and blood of the same cohort of mice, which allowed us to compare differences in the levels of individual cytokines as well as correlations among cytokines and 99mTc-DTPA uptake. We found that only the three-injection dose of LPS induced significant BBB disruption in all sexes and strains. The treatment, strain, and sex, as well as treatment-by- strain and treatment-by-sex interactions significantly contributed to the variance. The mean brain/serum ratios of 99mTc-DTPA in the three-injection LPS group were ranked CD-1 male < CD-1 female < C57 male < C57 female. There were significant sex and strain differences in cytokine profiles in brain and blood, and pro-inflammatory cytokines and chemokines in brain were most strongly correlated with 99mTc-DTPA brain/serum ratios.

## Introduction

The blood-brain barrier (BBB) is an important interface for neuroimmune communication and plays a multifaceted role in orchestrating the brain’s response to systemic inflammatory insults [1, 2]. One widely studied aspect of BBB dysfunction during inflammation is BBB disruption, which is the non-specific leakage of circulating molecules into the brain. BBB disruption is primarily attributed to pathophysiological changes in brain endothelial cells such as tight junction deficiencies, increased vesicular activities, and/or degeneration [2, 3]. A common method used to assess BBB disruption involves measuring the leakage of circulating tracers into the brain that are ordinarily mainly confined to the vascular space, and so whose uptake reflects the net effect of all pathophysiological changes at the BBB that facilitate tracer entry into the CNS. BBB disruption can cause or exacerbate CNS damage and neuroinflammation during a systemic inflammatory response, owing to leakage of circulating molecules into the brain that may stimulate immune activities in resident CNS cells [4, 5], or adversely impact neuronal function and survival [6, 7]. Cytokines are important mediators of BBB dysfunction in response to inflammatory insults [8], and may affect the BBB from the brain or blood compartment [9].

Lipopolysaccharide (LPS) is a prototypical stimulator of the innate immune system and has routinely been used to study the response of the BBB to systemic inflammation. Systemic elevations in LPS have also been associated with diseases with CNS sequalae such as hepatic encephalopathy[10], HIV infection [11], and metabolic syndrome [12]. It is appreciated that sex differences can influence the inflammatory response to acute insults, such as LPS. For example, resident immune cell populations and their receptors differ in the peritoneal and pleural cavities of male and female mice, which is in part influenced by estrogens [13]. Hypothermia, which is a CNS-mediated physiological response to LPS in mice, differs between males and females [14]. Sex differences in the immune response to infection can also influence prognostic outcomes. Data in humans and animal models suggest that males are generally more vulnerable to bacterial sepsis [15, 16], owing in part to differences in cytokine response patterns. In general, males exhibit higher levels of pro-inflammatory and Th1 cytokines, whereas females have higher levels of anti-inflammatory and Th2 cytokines [15, 16]. Recently, it has been shown that female C57BL6 mice are protected from BBB disruption following intraperitoneal LPS injections vs. male mice, which exhibit significant BBB disruption to Evan’s blue dye 4 hours but not 24 hours post-injection [17]. However, our group and others have found that BBB disruption to both small (14C sucrose and sodium fluorescein) and large (131I-albumin) molecular weight tracers is maximal at 24 hours post-LPS injection in male CD-1 mice [3, 4]. Therefore, we considered the possibility that genetic influences as reflected in strain-specific differences could affect the response of the BBB to LPS.

Genetic effects on physiological responses to LPS have been reported previously [18–21], and sexually dimorphic responses to LPS can vary by mouse strain [14]. Therefore, we felt that a systematic comparison was needed to determine whether sex-specific responses of the BBB to LPS could vary by differences in genetic background. We chose to compare C57BL6 mice and CD-1 mice since these two mouse strains are widely used in functional studies of the BBB [22]. We also compared brain and blood cytokine profiles in both sexes and strains due to their established contributions to BBB disruption and neuroinflammation.

## Materials and methods

### Animals

All mice were treated in accordance with NIH Guidelines for the Care and Use of Laboratory Animals in an AAALAC-accredited facility and approved by the Institutional Animal Care and Use Committee of the VA Puget Sound Health Care System (Protocol number 0909). Male and female CD-1 mice at 7 weeks of age were purchased from Charles River, and male and female C57BL6/J (C57) mice were purchased at 7 weeks of age from Jackson Laboratories. All mice were allowed to adapt for 1–2 weeks following shipment and were studied at 8–10 weeks of age. Mice were kept on a 12/12-h light/dark cycle with ad libitum food and water. Power analysis was used to determine the sample size needed to detect a 100 percent increase in tracer levels, which we have observed previously for ^14^C sucrose with LPS treatment [3]. 40 mice were tested in each sex and strain, amounting to a total of 160 mice for this study.

### Lipopolysaccharide (LPS) treatments

There were four treatment groups for each mouse sex and strain: LPS from Salmonella typhimurium (Sigma Aldrich, St. Louis, MO, USA) was dissolved in sterile normal saline for injections. We used three LPS treatment regimens. The first is an intraperitoneal (i.p.) three-injection regimen of 3mg/kg at t = 0, 6, and 24 hours. We have found that this regimen robustly and consistently disrupts the BBB to larger vascular space markers like albumin (MW = 66.5 kDa) in male CD-1 mice, as well as the smaller marker 14C-sucrose (340 Da) [3]. The second and third regimens are single i.p. injections of LPS at 3mg/kg or 0.3mg/kg, which were found to more moderately induce BBB disruption to 14C-sucrose, or to not induce detectable BBB disruption in male CD-1 mice, respectively [3]. The selections of these dosing regimens allow for comparison to previously published data in CD-1 male mice [3]. Control mice received three intraperitoneal (i.p.) injections of sterile normal saline (N.S.), given at t = 0, 6, and 24 hours. All mice were studied 28 hours after the first injection. The first LPS injections were always given in the morning, between the hours of 8:00 and 11:00. Mice were evaluated in 5 identical studies that spanned 11 days, and 2–4 mice from each treatment group from each sex and strain were used in each study, with the exception of one day in which mice from the female 0.3mg/kg single LPS injection group (see below) were reassigned to the 3-injection LPS groups since some mice died as a result of treatment or anesthesia prior to being studied.

### Evaluation of blood-brain barrier disruption

Mice were anesthetized with i.p. urethane, and 28 hours ± 20 minutes after the first LPS or saline injection, mice were injected in the jugular vein with 2 million CPM of 99mTc-DTPA (GE Healthcare, Boston, MA, USA) (487 Da) diluted in 1% bovine serum albumin (BSA) in lactated Ringer’s solution. After a circulation time of 20 minutes, arterial blood was collected from a cut in the descending abdominal aorta. The vascular space of the brain was washed free of blood by opening the thorax, clamping the descending thoracic aorta, severing both jugular veins, and perfusing 20 ml of lactated Ringer’s solution through the left ventricle of the heart in less than 1 min. After washout, the mouse was immediately decapitated and the whole brain was removed, cut along the sagittal suture, and the hemispheres were weighed. One hemisphere was counted in a Wizard2 2470 automatic gamma counter (Perkin Elmer, Waltham, MA, USA), and the other was frozen on dry ice. Serum was obtained by centrifuging the blood for 10 min at 2000g at 4 C and transferring to a clean tube. Two μl serum was counted on a gamma counter, and the remainder was aliquoted and frozen on dry ice. Frozen samples were stored at -80 C for 1 week prior to subsequent processing and analysis.

### Protein extraction from brain tissues

Frozen brain hemispheres were homogenized in buffer containing 10mM HEPES, 1.5mM MgCl2, 10mM KCl, 1mM dithiothreitol, and 1/100 dilutions of protease inhibitor (Sigma Aldrich, St. Louis, MO, USA) and phosphatase inhibitor (Sigma Aldrich, St. Louis, MO, USA) cocktails. Triton X-100 was added to the homogenate at a final concentration of 0.1%, and the samples were vortexed for 20 seconds, followed by centrifugation at 20,000g at 4 C for 10 minutes. The supernatants were aliquoted, and stored frozen at -80 C prior to cytokine measurements.

### Cytokine measurements

A panel of 23 cytokines (interleukin (IL)-1α; IL-1β; IL-2; IL-3; IL-4; IL-5; IL-6; IL-9; IL-10; IL-12(p40); IL-12(p70); IL-13; IL-17; eotaxin (CCL11); granulocyte colony-stimulating factor (G-CSF); granulocyte-macrophage colony-stimulating factor (GM-CSF); interferon (IFN)-γ; keratinocyte chemoattractant (KC) (CXCL1); monocyte chemoattractant protein (MCP)-1 (CCL2); macrophage inflammatory protein (MIP)-1α (CCL3); MIP-1β (CCL4); regulated on activation, normal T cell expressed and secreted (RANTES; CCL5) and tumor necrosis factor (TNF)-α) were measured in serum and brain protein extracts using a murine Bio-Plex Pro assay kit (Bio-Rad Laboratories, Inc.; Hercules, CA, USA). Serum samples were diluted by adding 15ul serum to 100ul of sample diluent provided with the kit. Serum standards were diluted in the standard diluent provided with the kit. Brain samples were diluted by adding 40ul of protein extract to 100ul of sample diluent provided in the kit. Brain standard diluent was prepared by adding 2 parts brain homogenization buffer with 0.1% Triton X-100 and 1.5% bovine serum albumin (BSA) to 5 parts sample diluent provided with the kit. Plates were processed according to the kit procedures, and read on a Bio-Plex 200 (Bio-Rad Laboratories, Inc.; Hercules, CA).

### Statistical analyses

Three-way ANOVAs were conducted to assess the overall contributions of treatment, sex, strain, and interactions on the variance. Bonferroni multiple-comparisons tests were conducted to evaluate significant differences in group means. To improve statistical power, we limited comparisons to those which evaluated mean differences in a single parameter (i.e. treatment, sex, or strain). For example, we compared C57 females treated with 3mg/kg LPS to CD-1 females, but not CD-1 males treated with the same dose of LPS. Equal group numbers were required to conduct three-way ANOVAs, and so 8 mice per group were evaluated even though some groups had 9 or 10 mice. Unequal numbers were due to premature death as a result of LPS treatment (see results), or anesthetic. Sample exclusion was conducted by rank-ordering the data in each group and dropping either the highest and lowest value (if n = 10) or the median value (if n = 9). Spearman correlations of 99mTc-DTPA vs. serum or brain cytokines were performed in a single analysis. All data were analyzed using Graphpad Prism 7 (GraphPad Software, San Diego, CA, USA)

## Results

### LPS effects on survival, weight loss, and spleen weights

**Fig 1:**
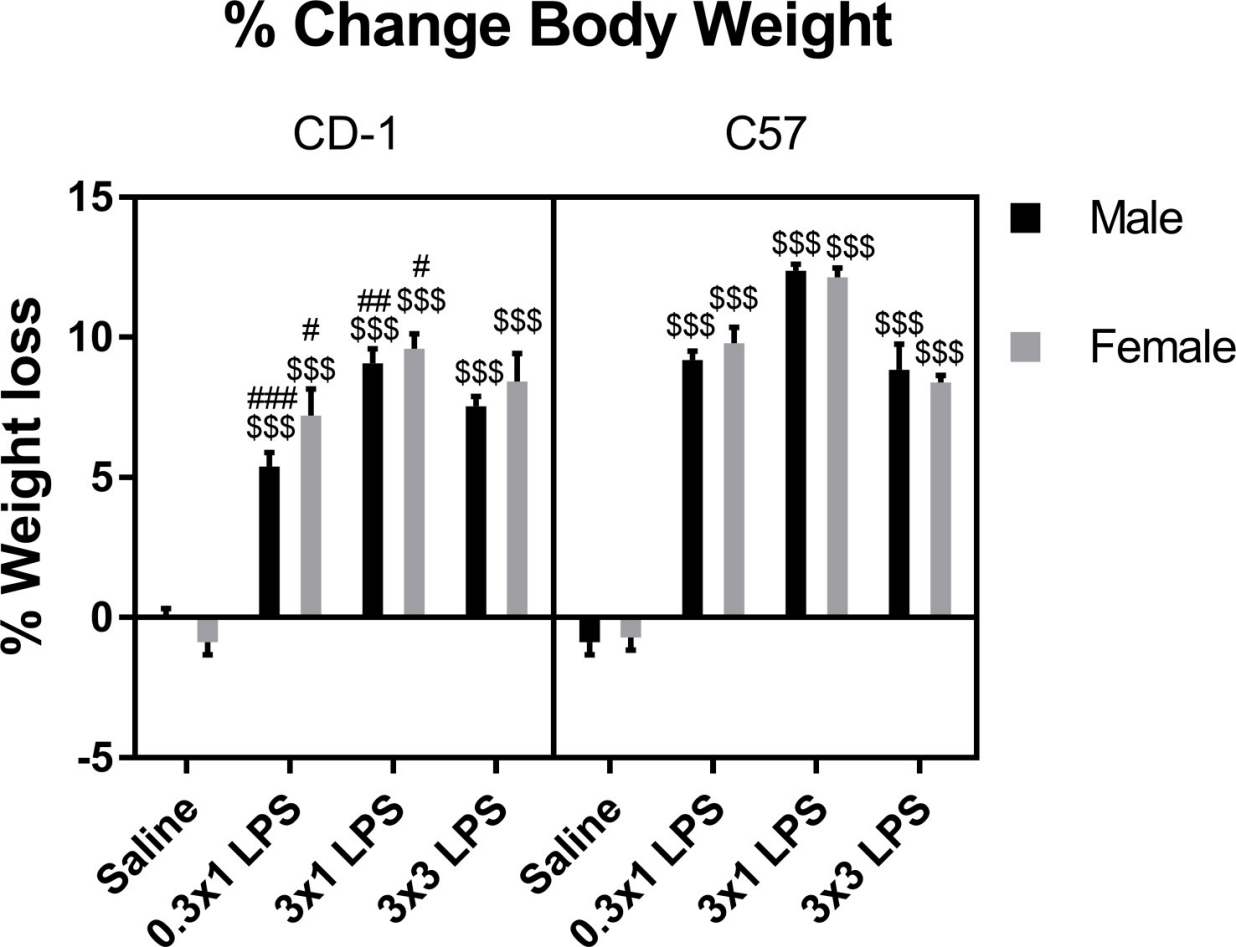
Effects of LPS on percent change in body weight. Data are presented as means ± SEM, n = 8 per group. $ $ $p < 0.001 vs. saline treatment within the same sex and strain. #p < 0.05, ##p < 0.01, ###p < 0.001 vs. C57 within the same treatment group and sex.

We first assessed effects of LPS on gross physiological parameters. Survival in both sexes and strains was 100% for saline and LPS single injection regimens. In the three-injection regimen of LPS, the death rate was 0/10 for CD-1 males, 1/12 for CD-1 females, 1/10 for C57 males, and 2/12 for C57 females. Significant reductions in body weight were apparent in all mice at each LPS dose, and CD-1 males had lower weight loss vs C57 males following single but not repeated LPS injections (Fig 1). There was no significant difference in weight loss at any LPS dose when comparing CD-1 and C57 females, or sexes within either strain of mouse. Three-way ANOVA of all groups showed that treatment was the major contributor to variation among groups. Strain, and treatment-strain interaction effects also significantly contributed to the variation (Table 1).

**Table 1:** Three-way ANOVA analysis of percent change in body weight.

Source of Variation	% of total variation	P value
Treatment	83.94	<0.0001
Strain	2.879	<0.0001
Sex	0.1018	0.2827
Treatment x Strain	2.571	<0.0001
Treatment x Sex	0.3699	0.2433
Strain x Sex	0.08921	0.3144
Treatment x Strain x Sex	0.2688	0.3842

### LPS effects on BBB disruption

Next, we determined the brain uptake of circulating 99mTc-DTPA to indicate whether disruption to the BBB occurs. ^99m^Tc-DTPA is a small molecular weight marker that has been used to measure BBB disruption in rodents and humans via SPECT imaging [23–26]. ^99m^Tc-DTPA has a molecular weight of 487 Da, which is comparable to that of ^14^C-sucrose (342.3 Da) that we have used in previous studies [3, 27]. Because it is a gamma emitter, ^99m^Tc-DTPA can be sensitively and quantitatively detected by a gamma counter. Its short half-life also permits subsequent assessment of biochemical parameters, such as cytokines, which allows for intra-individual matching for correlation analysis.

**Fig 2:**
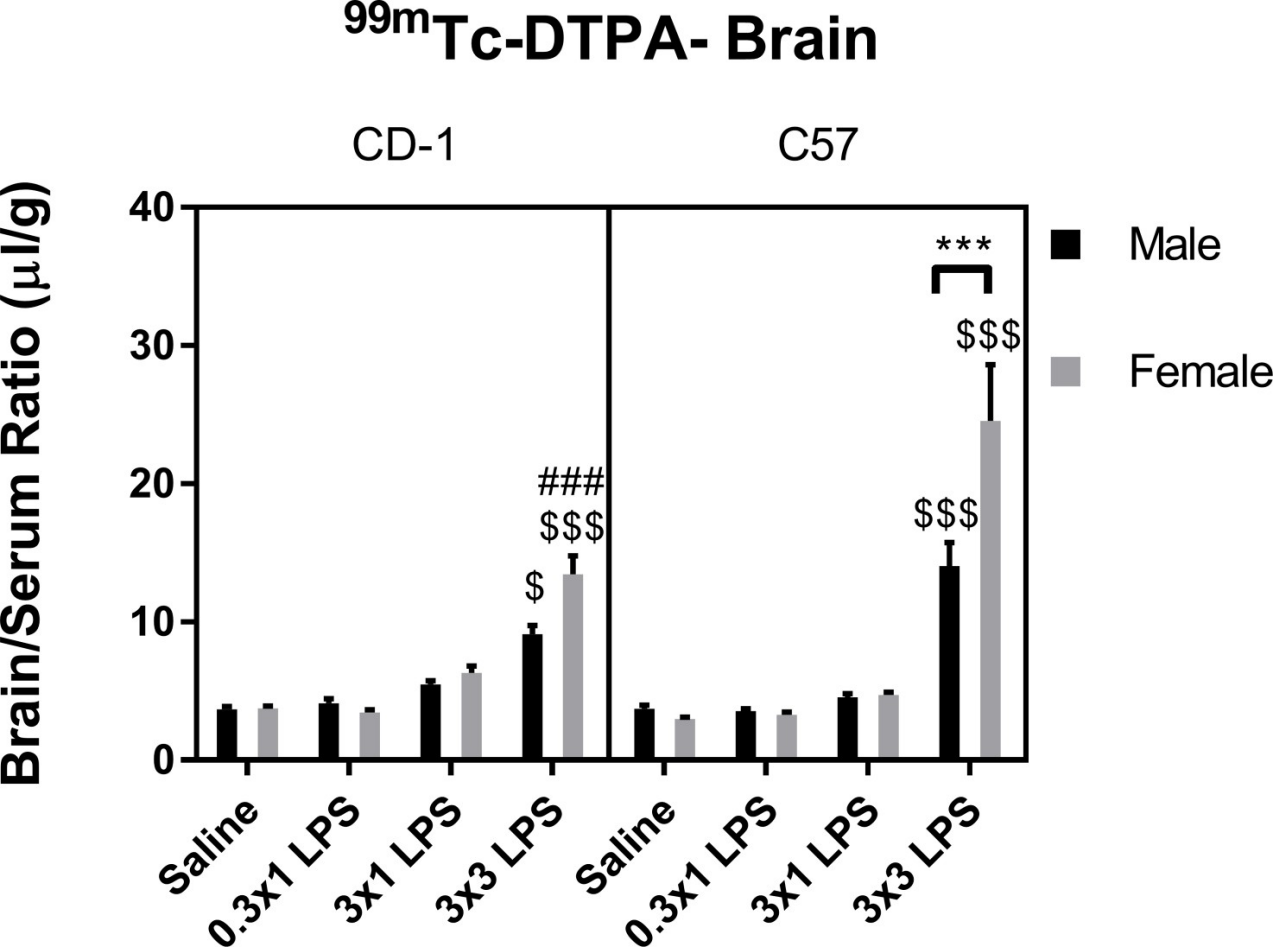
Effects of LPS on brain/serum ratios of ^99m^Tc-DTPA. Data are presented as means ± SEM, n = 8 per group. $p < 0.05, $ $ $p < 0.001 vs. saline treatment within the same sex and strain. ###p < 0.001 vs. C57 within the same treatment group and sex. ***p < 0.001 vs. groups indicated, within the same strain and treatment.

**Fig 3:**
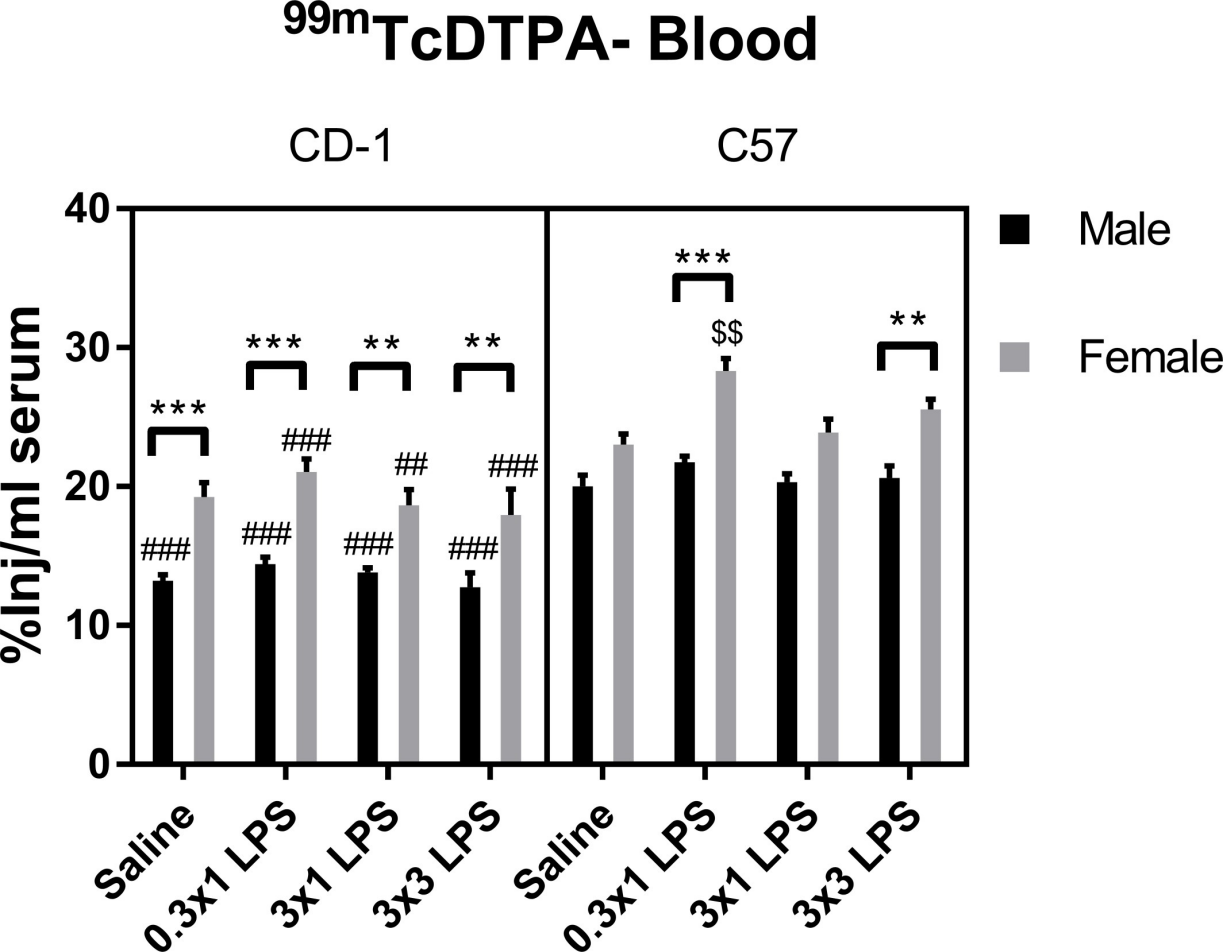
Effects of LPS on serum levels of ^99m^Tc-DTPA. Data are presented as means ± SEM, n = 8 per group. $ $p < 0.01 vs. saline treatment within the same sex and strain. ##p < 0.01, ###p < 0.001 vs. C57 within the same treatment group and sex. **p, 0.01, ***p < 0.001 vs. groups indicated, within the same strain and treatment.

We found that ^99m^Tc-DTPA levels in brain were only significantly increased vs. saline control by the three-injection regimen of LPS in both sexes and strains (Fig 2). Three-way ANOVA of all groups showed that treatment contributed to the majority of variation among groups. There were also significant effects of sex, strain, and treatment-sex and -strain interactions (Table 2). Multiple comparisons tests showed that C57 females had significantly worse BBB disruption in response to three injections of LPS vs. C57 males and CD-1 females. However, there were not significant strain differences observed in males, or sex differences observed in CD-1 mice at the BBB-disrupting LPS regimen in our post-hoc tests. No sex or strain differences in brain uptake of ^99m^Tc-DTPA were apparent in the single LPS injection regimens or in saline controls. Because our brain uptake measures of ^99m^Tc-DTPA include normalization to blood levels, we also evaluated concentrations of ^99m^Tc-DTPA in blood (Fig 3). ^99m^Tc-DTPA concentrations in blood differed between sexes and strains (Table 3), which was expected due to sex and strain differences in body weight (S1 Fig). Treatment-associated differences in mean ^99m^Tc-DTPA blood levels were not apparent, except in female C57 mice at the lowest LPS single dose of 0.3mg/kg. Therefore, LPS treatments that disrupted the BBB did not significantly affect blood concentrations of ^99m^Tc-DTPA in any sex or strain.

**Table 2:** Three-way ANOVA analysis of brain/serum ratios of ^99m^Tc-DTPA.

Source of Variation	% of total variation	P value
LPS dose	56.91	<0.0001
Strain	1.358	0.0122
Sex	1.899	0.0032
LPS dose x Strain	8.542	<0.0001
LPS dose x Sex	6.386	<0.0001
Strain x Sex	0.2352	0.2915
LPS dose x Strain x Sex	1.216	0.1278

**Table 3:** Three-way ANOVA analysis of serum levels of ^99m^Tc-DTPA.

Source of Variation	% of total variation	P value
Treatment	4.083	0.0004
Sex	43.54	<0.0001
Strain	26.39	<0.0001
Treatment x Sex	1.022	0.1861
Treatment x Strain	0.8681	0.2509
Sex x Strain	0.3429	0.2027
Treatment x Sex x Strain	0.3605	0.6323

### LPS effects on blood and brain cytokines

**Fig 4:**
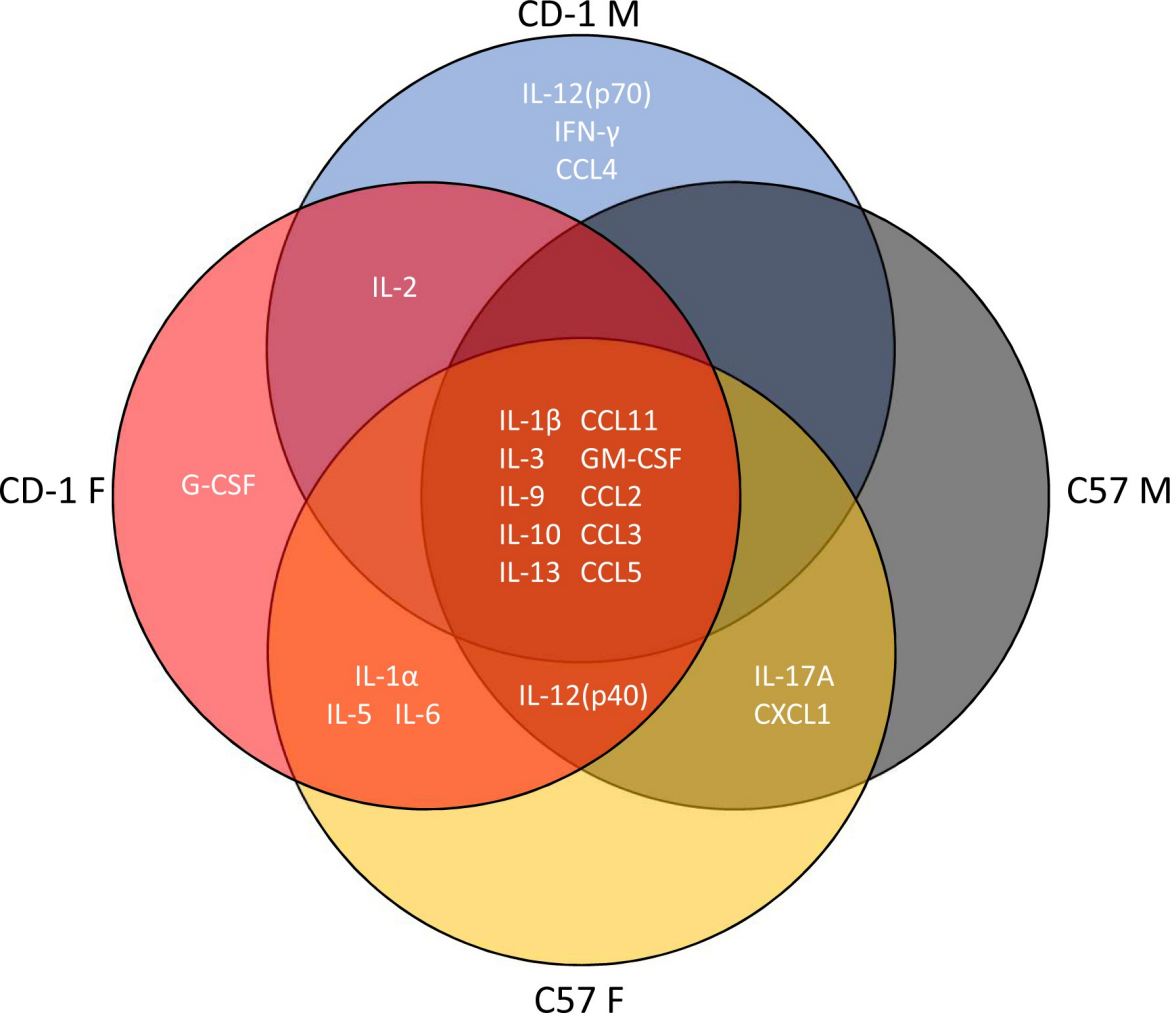
Venn diagram of significant cytokine elevations in serum following LPS treatment according to sex and strain.

**Fig 5:**
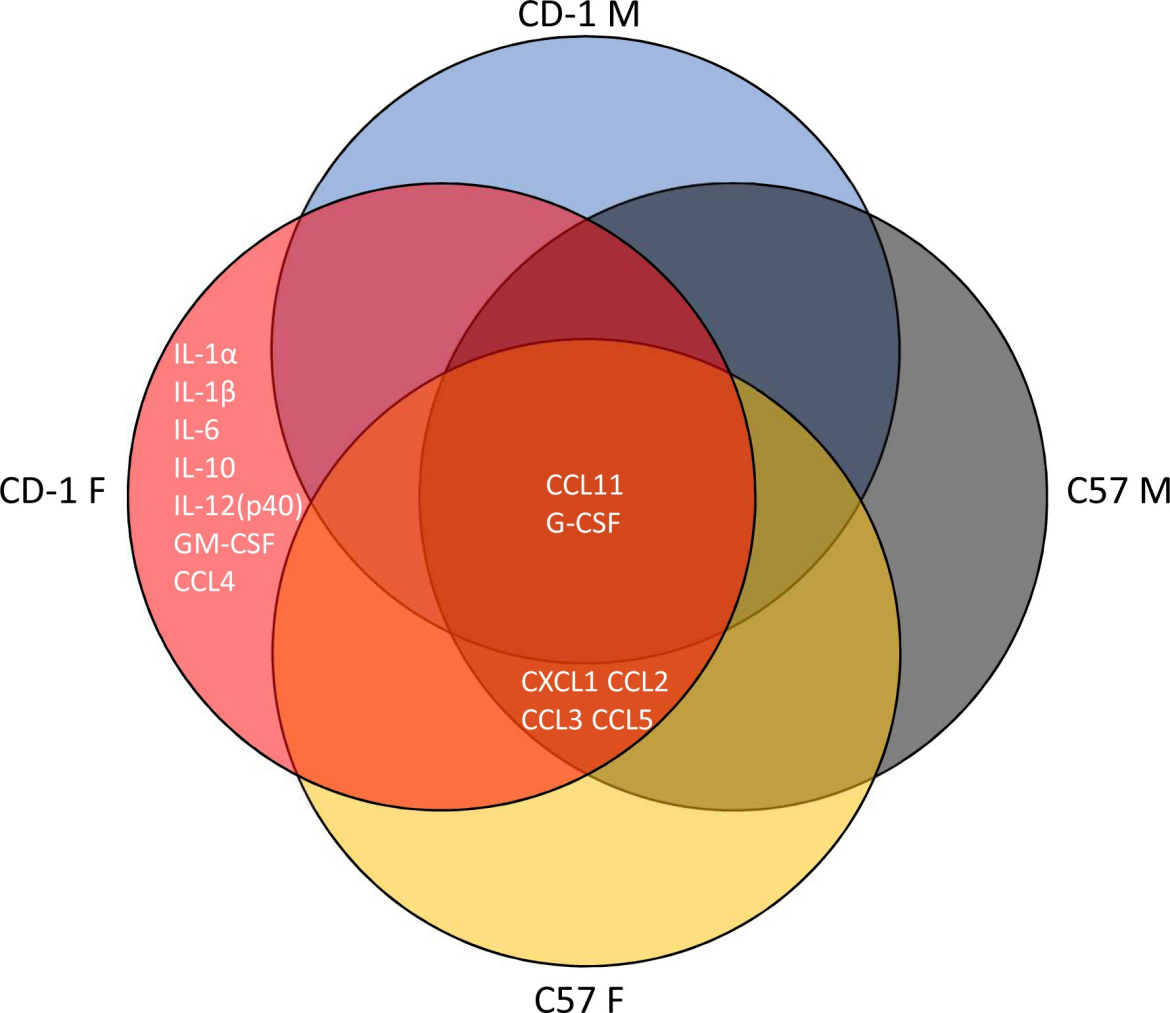
Venn diagram of significant cytokine elevations in brain following LPS treatment according to sex and strain.

We next compared cytokine profiles in mice treated with saline or the triple dose of LPS to examine associations of cytokine expression patterns with BBB disruption. Three-way ANOVA and multiple comparisons test results for each individual cytokine are shown in S1–S13 Figs. These results are summarized in Fig 4 and Table 4 for blood and Fig 5 and Table 5 for brain. There were no significant sex/strain differences in brain or serum levels of any cytokine in the saline-treated groups. In serum, LPS significantly increased levels of 21/23 cytokines in at least one sex or strain category (summarized in Fig 4 and Table 4). LPS significantly increased IL-1β, IL-3, IL-9, IL-10, IL-13, CCL-11, GM-CSF, CCL2, CCL3, and CCL5 in all sexes and strains. IL-12(p40) was significantly upregulated in all groups except CD-1 males. IL-5, IL-1α, and IL-6 were significantly upregulated by LPS in females only, IL-2 was significantly upregulated in CD-1 mice, and IL-17A and CXCL1 were significantly upregulated in C57 mice. LPS significantly upregulated IL-12(p70), IFN-γ, and CCL4 in CD-1 males only, and G-CSF in CD-1 females only. There were also significant differences in LPS-induced elevations of serum cytokines among sexes and strains (summarized in Table 4). IL-5, IL-10, CCL11, and CXCL1 had significantly higher levels in LPS-treated C57 females vs C57 males or CD-1 females. IL-10 and IL12(p40) were significantly lower in CD-1 males vs. C57 males. CD-1 males also had lower IL12(p40) vs. CD-1 females. Significant strain-only differences included IL-12(p70), where male and female CD-1 mice had higher levels than C57 mice of the same sex. Female CD-1 mice had higher levels than GM-CSF vs. C57 females, and C57 females had higher levels of CCL3 vs. CD-1 females.

**Table 4:** Significant changes in serum cytokines according to sex and strain following LPS treatment.

Cytokine	Increased with LPS	Strain differences	Sex differences
IL-1α	CD-1 F**, C57F***		
IL-1β	CD-1 M***, CD-1F***, C57M***, C57F***		
IL-2	CD-1M*, CD-1F**		
IL-3	CD-1 M***, CD-1F***, C57M***, C57F***		
IL-4		LPS Male (CD-1)*	
IL-5	CD-1F***, C57F***	LPS Female (C57)*	LPS C57 (F)*
IL-6	CD-1F*, C57F*		
IL-9	CD-1 M***, CD-1F***, C57M***, C57F***		
IL-10	CD-1 M**, CD-1F***, C57M***, C57F***	LPS Male (C57)***	LPS C57 (F)*
		LPS Female (C57)***	
IL-12(p40)	CD-1F***, C57M***, C57F***	LPS Male (C57)***	LPS CD-1 (F)**
		LPS Female (C57)***	
IL-12(p70)	CD-1 F**	LPS Female (CD-1)**	
		LPS Male (CD-1)*	
IL-13	CD-1 M**, CD-1F***, C57M***, C57F***		
IL-17A	C57M*, C57F***		
CCL11	CD-1 M***, CD-1F***, C57M**, C57F***	LPS Female (C57)***	LPS C57 (F)***
G-CSF	CD-1F**		
GM-CSF	CD-1 M**, CD-1F***, C57M*, C57F**	LPS Female (CD-1)**	
IFN-γ	CD-1M*		
CXCL1	C57M***, C57F***	LPS Female (C57)***	LPS C57 (F)***
CCL2	CD-1 M***, CD-1F***, C57M*, C57F***		
CCL3	CD-1 M***, CD-1F***, C57M***, C57F***	LPS Female (C57)*	
CCL4	CD-1M***		
CCL5	CD-1 M***, CD-1F***, C57M***, C57F***		
TNF-α		LPS Female (CD-1)***	

*p < 0.05

**p < 0.01

***p < 0.001. Strain or sex in parenthesis indicates the group with the higher mean cytokine concentration.

**Table 5:** Significant changes in brain cytokines according to sex and strain following LPS treatment.

Cytokine	Increased with LPS	Strain differences	Sex differences
IL-1α	CD-1F***		LPS CD-1 (F)**
IL-1β	CD-1F***	LPS F (CD-1)**	
IL-6	CD-1F***		LPS CD-1 (F)**
IL-10	CD-1F***		
IL-12(p40)	CD-1F***	LPS F (CD-1)**	
CCL11	CD-1 M***, CD-1F***, C57M**, C57F***		
G-CSF	CD-1 M*, CD-1F***, C57M**, C57F***		LPS CD-1 (F)**
GM-CSF	CD-1F***		LPS CD-1 (F)*
CXCL1	CD-1F***, C57M**, C57F***		LPS CD-1 (F)***
CCL2	CD-1F***, C57M**, C57F***		
CCL3	CD-1F***, C57M**, C57F***		LPS CD-1 (F)*
CCL4	CD-1F*		
CCL5	CD-1F***, C57M**, C57F*		LPS CD-1 (F)*

*p < 0.05

**p < 0.01

***p < 0.001.

Strain or sex in parenthesis indicates the group with the higher mean cytokine concentration.

In brain, CCL11 and G-CSF were significantly increased by LPS in all sexes and strains. All groups but CD-1 males had significant upregulation of CXCL1, CCL2, CCL3, and CCL5. IL-1α, IL-1β, IL-6, IL-10, IL-12(p40), GM-CSF, and CCL4 were significantly upregulated by LPS in CD-1 females only (Fig 5). Sex or strain-specific differences were observed for IL-1α, IL-1β, IL-6, IL-12(p40), G-CSF, GM-CSF, CXCL1, CCL3, and CCL5, with CD-1 females having the highest expression in all comparisons (Table 5).

### Correlations of blood and brain cytokines with BBB disruption

**Fig 6:**
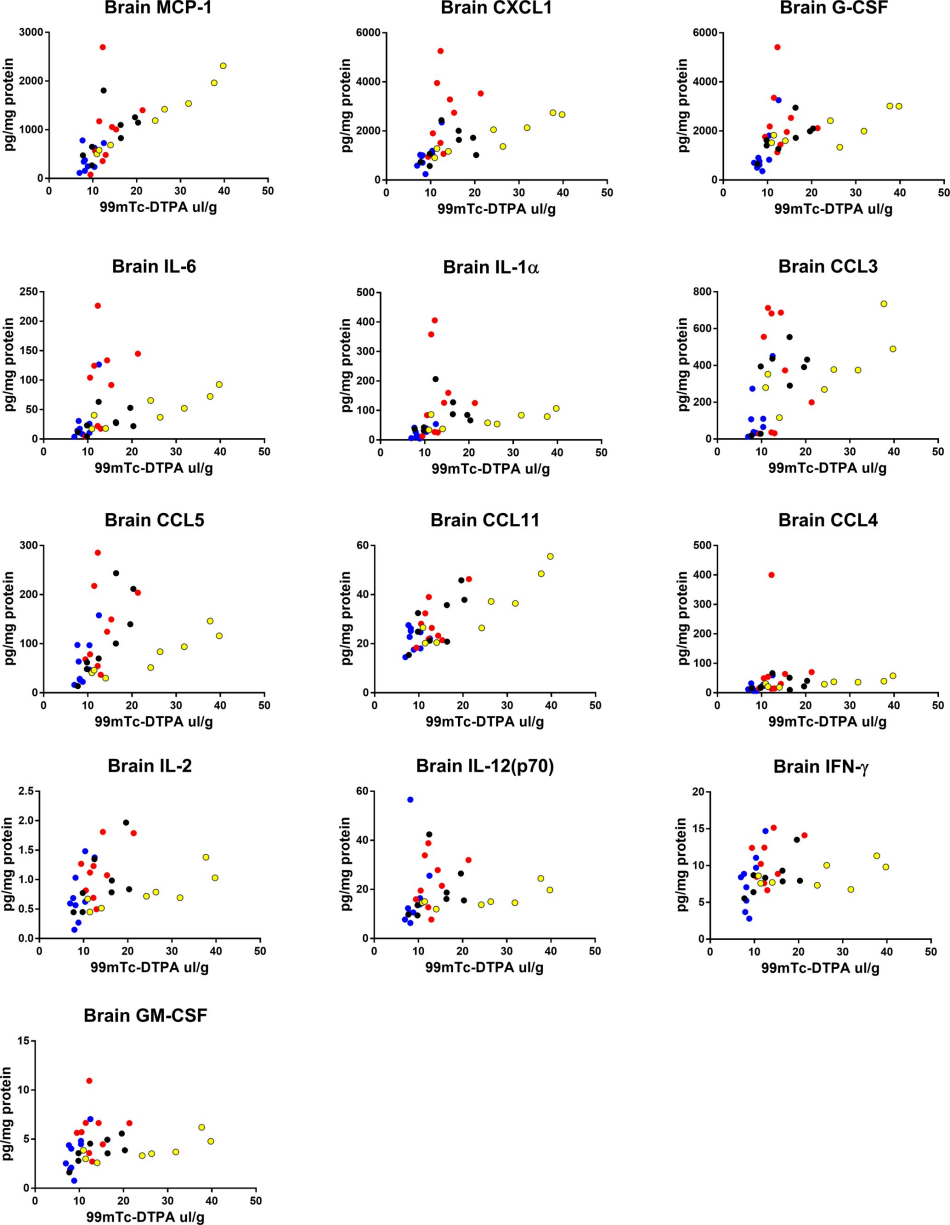
Correlations of brain cytokines with brain/serum ratios of 99mTc-DTPA. Data from the 3 injection LPS groups for all sexes and strains were pooled and correlated for each detectable cytokine in brain. Graphs are showing statistically significant Spearman correlations. Blue dots = male CD-1, black dots = male C57, red dots = female CD-1, yellow dots = female C57.

**Fig 7:**
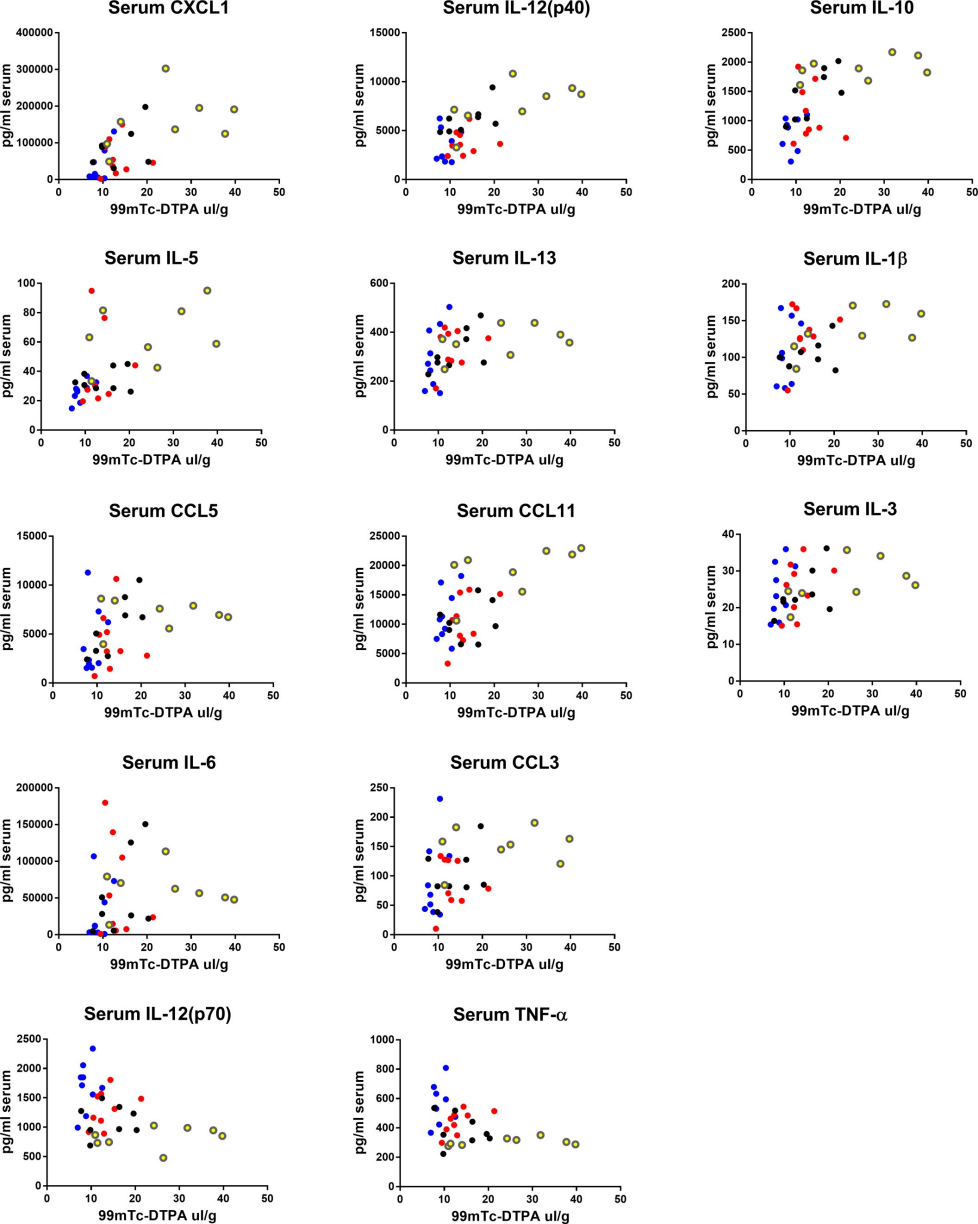
Correlations of serum cytokines with brain/serum ratios of ^99m^Tc-DTPA. Data from the 3 injection LPS groups for all sexes and strains were pooled and correlated for each detectable cytokine in brain. Graphs are showing statistically significant Spearman correlations. Blue dots = male CD-1, black dots = male C57, red dots = female CD-1, yellow dots = female C57.

We next determined which cytokines in blood or brain correlated with ^99m^Tc-DTPA uptake in brains in all sexes and strains within the LPS-only group. Spearman analysis was used to assess relations since many relations appeared to be non-linear. We found that the strongest positive correlations with our marker of BBB disruption tended to be brain cytokines that are associated with innate immune cell trafficking, and pro-inflammatory cytokines and growth factors also in brain (Table 6). Notably, brain cytokine relations to ^99m^DTPA tended to diverge (Fig 6), showing an overall trend that brain cytokines predict BBB disruption well in C57 females, but poorly in CD-1 females. Serum correlations were less divergent, and showed positive relationships of increased pro-inflammatory cytokines and chemokines (Fig 7), but also anti-inflammatory cytokines with DTPA. Serum IL-12p70 and TNF-α negatively correlated with BBB disruption.

**Table 6:** Correlations of 99mTc-DTPA brain/serum ratios with brain and serum cytokines in LPS-treated mice.

Cytokine	Tissue	Spearman r	p-value
CCL2	Brain	0.7953	<0.0001
CXCL1	Brain	0.7051	<0.0001
CXCL1	Serum	0.6859	<0.0001
G-CSF	Brain	0.673	<0.0001
IL-6	Brain	0.6345	<0.0001
IL-1α	Brain	0.6211	<0.0001
IL-12(p40)	Serum	0.6086	0.0001
CCL3	Brain	0.5979	0.0002
IL-10	Serum	0.5951	0.0002
CCL5	Brain	0.5869	0.0003
IL-5	Serum	0.5792	0.0003
CCL11	Brain	0.5752	0.0004
CCL4	Brain	0.5435	0.0009
IL-13	Serum	0.5181	0.0017
IL-1β	Serum	0.4951	0.0029
CCL5	Serum	0.4903	0.0032
IL-2	Brain	0.479	0.0042
CCL11	Serum	0.4564	0.0067
IL-3	Serum	0.4497	0.0076
IL-6	Serum	0.4429	0.0087
IL-12(p70)	Brain	0.435	0.0101
CCL3	Serum	0.427	0.0118
CCL2	Serum	0.4188	0.0137
IFN-γ	Brain	0.3708	0.0309
GM-CSF	Brain	0.3574	0.038
IL-12(p70)	Serum	-0.3498	0.0426
TNF-α	Serum	-0.4026	0.0182

## Discussion

Overall, our main findings show that there are sex and strain differences in the magnitude of LPS-induced BBB disruption, and that BBB disruption positively correlates with levels of pro-inflammatory cytokines and chemokines in brain. For all mouse sexes and strains, significant changes in BBB disruption to ^99m^Tc-DTPA were only apparent following the three-injection paradigm of 3mg/kg LPS. Compared to ^14^C-sucrose, which we have previously used as a marker for BBB disruption [3], ^99m^Tc-DTPA appears to have lower brain uptake in control animals and following LPS treatment, despite having a molecular weight that is only slightly larger than ^14^C-sucrose. It is possible that other properties of DTPA related to its chemical structure, such as hydrodynamic radius, may contribute to its lower levels of brain uptake. Although minor increases in ^99m^Tc-DTPA brain uptake were apparent at the single 3mg/kg dose of LPS, these were not statistically significant vs. control animals. Further, there was a lack of apparent sex/strain differences in ^99m^Tc-DTPA brain uptake with single dosing, suggesting that sex and strain-dependent differences in BBB disruption are more predominant when systemic inflammation is severe.

Our results also show that the sexually dimorphic response of the BBB to LPS is strain-dependent. Although CD-1 females had an increased mean uptake of ^99m^Tc-DTPA with LPS treatment in comparison to the other three groups, there were no significant differences between CD-1 males and females. However, C57 females had significantly more LPS-induced BBB disruption than C57 males. C57 females also had more BBB disruption vs. CD-1 females. Previously, it was shown in C57BL6 mice that estrogen protects female mice from BBB disruption induced by LPS [17]. Our results appear to conflict with these findings. However, there were notable methodological differences that include the use of a different LPS serotype, as well as a difference in the tracer used to evaluate BBB disruption, which was Evan’s blue. In principle, Evan’s blue levels in the brain proxy the brain entry of albumin, which is an abundant serum protein that does not cross the intact BBB. Methodological issues associated with the use of Evan’s blue have been reviewed elsewhere [28, 29], warranting careful consideration of its use to assess BBB disruption during inflammatory conditions in which serum albumin levels can decrease [30] and potentially influence results. Another notable difference in our results vs. those of Maggioli et al is the timing of BBB disruption following LPS treatment. In our study, we assessed BBB disruption 28 hours following the first injection of LPS in all groups. We chose this time point based on our group’s previous findings in male CD-1 mice that BBB disruption to ^14^C sucrose was evident at 24 hours, but not 4 hours following a single LPS injection of 3mg/kg [3]. Other groups in addition to ours have also found BBB disruption to tracers such as sodium fluorescein and ^125^I-albumin are significantly increased 24 hours post-LPS injection [3, 4, 31]. In contrast, results from Maggioli et al showed that brain levels of Evan’s blue were significantly increased at 4 hours post-LPS and returned to baseline by 24 hours [17], which was also shown by another group [32]. Therefore, it is possible that processes governing brain uptake of Evan’s blue at 4–6 hours post-LPS injection could be distinct from those which regulate BBB disruption at later time points or following a multiple LPS injection paradigm. It is presently unclear whether the increased vulnerability of C57 female mice to LPS-induced BBB disruption is estrogen-mediated, but is a logical future direction of this study.

We observed sex- and strain-specific patterns of cytokines in blood and brain following LPS treatment. In blood, C57 females had the highest levels of the neutrophil chemokines CXCL1 and CCL3, the eosinophil chemokine CCL11, the pro-inflammatory cytokine IL-12p40, the anti-inflammatory cytokine IL-10, and the Th2 cytokine and eosinophil stimulator IL-5. To our knowledge, none of these cytokines have been assessed for BBB-disrupting activities, with the exception of CCL11, which has been shown not to cause BBB disruption [33]. However, each of these cytokines significantly correlated with BBB disruption to 99mTc-DTPA, which suggests that they may be peripheral indicators of BBB disruption, or contribute to BBB disruption, either directly or indirectly. In brain, CD-1 males tended to have lower levels of pro-inflammatory chemokines, suggesting that chemotaxis into or within the brain is also reduced. Overall, CD-1 females had the highest brain levels of pro-inflammatory cytokines, chemokines and growth factors. This was a surprising finding, since CD-1 females were more resistant to BBB disruption vs. C57 female mice. Further, the correlative relations of many brain cytokines with brain uptake of ^99m^Tc-DTPA were not apparent in CD-1 female mice. These findings may indicate that although CD-1 female mice have an exacerbated neuroinflammatory response to LPS, their BBB is more resistant to disruption.

Sepsis-associated encephalopathy (SAE) is one example of a pathophysiological neuroimmune interaction where BBB disruption can contribute to CNS dysfunction and damage. Male mice and humans are more vulnerable to sepsis than females: males have a higher risk of developing sepsis following trauma, septic shock is more prevalent in males vs. females with sepsis, and men have a greater mortality rate [16]. On the other hand, human females with septic encephalopathy were shown to be more likely to die than males [34], which could reflect a greater sensitivity of the BBB to inflammation in females. Interestingly, we found that more female mice died from LPS treatment vs. male mice. Although this finding seems contrary to observations in sepsis, it may be explained by the absence of overt infection in our model. One reason that females may be less vulnerable to sepsis than males is that their immune systems are able to more efficiently contain and clear pathogens [13]. However, in a study where humans were treated with LPS in vivo, females had greater elevations in blood leukocytes, C-reactive protein, and pro-inflammatory cytokines such as TNF-α and IFN-γ [35]. In the same study, it was found that females had more pronounced decreases in blood pressure and norepinephrine sensitivity than males. One limitation to our study is that we only measured terminal concentrations of cytokines and chemokines, and so we may have missed important relations of cytokines known to peak at earlier time points to BBB disruption [36]. However, our data generally agreed with human data that females had higher levels of cytokines and chemokines than males in blood and brain following LPS. Therefore, although females may be better able to fight off infections, data from our group and others also suggests that females may have exacerbated inflammatory responses vs. males when presented with the same dose of an immune stimulus.

## Conclusions

In conclusion, we have shown that the sex-dependent effects of LPS on the mouse BBB vary by strain. At 28 hours after the first exposure to LPS, C57 females had worse BBB disruption than C57 males, which was associated with higher levels of cytokines and chemokines in blood, but not brain. CD-1 female mice were more resistant to BBB disruption vs. C57 females, despite higher brain levels of cytokines and chemokines. Finally, we were able to identify cytokines and chemokines in blood and brain, which were the most strongly correlated with BBB disruption. Logical future directions of this work include investigations into sex-hormone differences in C57 vs. CD-1 female mice that could mediate their apparent differences in BBB sensitivity to LPS, and exploration of novel mechanisms by which the cytokines, chemokines, and growth factors that significantly correlated with 99mTc-DTPA uptake could mechanistically contribute to BBB disruption.

## Supporting information

S1 Fig Mouse body weights prior to treatment. (TIF)

S2 Fig Effects of sex and strain on serum IL-1α, IL-1β, IL-2, and IL-3 responses to LPS. (TIF)

S3 Fig Effects of sex and strain on serum IL-4, IL-5, IL-6, and IL-9 responses to LPS. (TIF)

S4 Fig Effects of sex and strain on serum IL-10, IL-12(p40), IL-12(p70), and IL-13 responses to LPS. (TIF)

S5 Fig Effects of sex and strain on serum IL-17A, CCL11, G-CSF, and GM-CSF responses to LPS. (TIF)

S6 Fig Effects of sex and strain on serum IFN-γ, CXCL1, CCL2, and CCL3 responses to LPS. (TIF)

S7 Fig Effects of sex and strain on serum CCL4, CCL5, and TNF-α responses to LPS. (TIF)

S8 Fig Effects of sex and strain on brain IL-1α, IL-1β, IL-2, and IL-3 responses to LPS. (TIF)

S9 Fig Effects of sex and strain on brain IL-4, IL-5, IL-6, and IL-10 responses to LPS. (TIF)

S10 Fig Effects of sex and strain on brain IL-12(p40), IL-12(p70), and IL-17A, and CCL11 responses to LPS. (TIF)

S11 Fig Effects of sex and strain on brain G-CSF, GM-CSF, IFN-γ, and CXCL1 responses to LPS. (TIF)

S12 Fig Effects of sex and strain on brain CCL2, CCL3, CCL4, and CCL5 responses to LPS. (TIF)

S13 Fig Effects of sex and strain on brain TNF-α responses to LPS. (TIF)
